# Association Between Quality of Life, Anxiety, and Pain Among Older Adults

**DOI:** 10.7759/cureus.85522

**Published:** 2025-06-07

**Authors:** Maria Massa, Marianna Mantzorou, Theodoula Adamakidou, Alexandra Koreli, Paraskevi Apostolara, Marianna Drakopoulou, Georgia Gerogianni

**Affiliations:** 1 Department of Nursing, Postgraduate Program "Community and Public Health Nursing", University of West Attica, Athens, GRC

**Keywords:** anxiety, comorbidities, older adults, pain, quality of life

## Abstract

Introduction: The quality of life of older adults is strongly affected by anxiety and chronic pain, which leads to psychological distress and reduced functionality. The purpose of this study was to investigate the association between quality of life, anxiety, and pain among older adults.

Methods: In this quantitative cross-sectional study, one hundred (n=100) participants with a mean age of 75.3 years (SD = 7.7 years) took part in the study. Quality of Life, Anxiety, and Pain were evaluated via i) the SF-12 Health Survey, ii) the State-Trait Anxiety Inventory (STAI), and iii) the Brief Pain Inventory (BPI), respectively, and a questionnaire about demographic characteristics. Pearson’s or Spearman’s correlation coefficients were used to explore the association of two continuous variables. Multiple linear regression analysis in a stepwise method was used with dimensions of quality of life as dependent variables.

Results: Both state and trait anxiety were significantly associated with poor mental health and quality of life (p<.001), while increased pain was significantly associated with low physical health (p<.001) and high state (p=.012) and trait anxiety (p<.001). Individuals who had been involved in manual work reported decreased physical health (p=.036) and poor mental health (p<.001). Those who lived alone had poor mental health (p=.009), while high trait anxiety was significantly associated with increased age (p=.043).

Conclusions: Early assessment of anxiety and pain can improve the physical and mental health of older adults and their overall quality of life. A multidisciplinary approach to pain management can prevent the deterioration of physical health among this population.

## Introduction

The quality of life of older adults depends on their perceived well-being, their functional ability to perform daily activities, their level of independent living [1], and their participation in social activities [2]. However, increased age includes a high risk of developing comorbid diseases [3], which lead to increased disability, loss of functional capacity, and decline in the overall quality of life among older adults [4]. Comorbid diseases among this population include chronic conditions such as dyslipidemia, hypertension, diabetes mellitus, coronary heart disease, stroke, chronic obstructive pulmonary disease, chronic kidney disease, cancer, mental disorders, and osteoarthritis [3].

Additionally, anxiety is a significant factor that negatively affects the quality of life of older adults [5], which can be deteriorated by an increase in age [6] due to medical and psychiatric disorders and living conditions [7]. Furthermore, the quality of life of older people is negatively affected by chronic pain, which leads to psychological distress and reduced functionality [8]. Chronic pain is a common symptom in individuals over 65 years old due to chronic conditions [9], leading to loss of mobility and independence, limitations in social relationships and activities [10], as well as anxiety and depression [9].

Although many studies have examined the quality of life, anxiety, or pain in the Greek population, few studies have explored the interrelationship between anxiety and pain and its impact on the quality of life among this specific group of older adults in Greece. These findings can help healthcare providers develop targeted strategies for monitoring and improving the mental and physical health of older adults in Greece and enhance the quality of their lives. The aim of this study was to investigate the association between quality of life, anxiety, and pain among older adults.

## Materials and methods

Study design

This study was a cross-sectional correlational study aiming to explore the association between quality of life, anxiety, and pain among older adults.

Study population

The study was conducted in Athens, Greece, with a conveniently selected sample of 100 older adults. The main researcher recruited participants who attended a primary health care center in Piraeus for a follow-up on various chronic conditions.

The selection criteria for participants were being 65 years old or older and understanding, speaking, and reading Greek. Exclusion criteria were insufficient language ability, age under 65 years old, cognitive deterioration, psychiatric disorders, and drug or alcohol abuse.

Out of the 130 older adults who were recruited in order to participate in the study, five had a cognitive impairment diagnosis according to their medical file and were excluded from the study. Moreover, 25 out of 125 participants who met the inclusion criteria refused to participate due to time constraints, so the response rate of the study was 80%. No drug or alcohol addicts or persons with diagnosed psychiatric disorders attended the health center during the period of the study so as to be approached for inclusion in the study.

Sampling technique and sample size

The sample size was calculated a priori. Using the software program G*Power (version 3.1.9.6, 2020), the authors opted for a 95% power in multivariate linear regression analysis with a total number of five predictors with an error level of α=0.05, able to detect an effect size of at least 0.2. This yielded a desired sample size of 105. Our final model, consisting of four predictors, would need 98 participants to cover the above requirements; therefore, our sample size was deemed adequate.

Data collection tools

The questionnaires SF-12 Health Survey, State-Trait Anxiety Inventory (STAI), and Brief Pain Inventory (BPI) were utilized for the evaluation of quality of life, anxiety, and pain, respectively, as well as a questionnaire regarding demographic characteristics.

SF-12 Health Survey

The 12-item Health Survey (SF-12) is used to assess physical and mental health. Four health concepts-physical functioning (PF), role physical (RP), role emotional (RE), and mental health (MH)-are measured using two items each, while the remaining domains-bodily pain (BP), general health (GH), vitality (VT), and social functioning (SF)-are assessed using a single item each. The SF-12 is a valid and efficient tool for assessing health-related quality of life in the Greek population [11].

State-Trait Anxiety Inventory (STAI)

The State-Trait Anxiety Inventory (STAI) is a well-established and widely used tool in clinical and research settings for measuring anxiety. Its development began in 1964 by C.D. Spielberger and R.L. Gorsuch, with the initial version, STAI Form X, released in 1970 [12]. A revision process started in 1979, leading to the release of the updated Form Y in 1985 [13]. The STAI consists of two separate subscales. The State Anxiety Scale (Form Y-1) includes 20 statements that assess how an individual feels “right now, at this moment.” The Trait Anxiety Scale (Form Y-2) includes 20 items measuring how the person feels “generally.”

Responses are rated on a four-point Likert scale (ranging from 1 to 4). For the State Anxiety Scale, higher scores on items 3, 4, 6, 7, 9, 12, 13, 14, 17, and 18 reflect greater anxiety. For the Trait Anxiety scale, items 22, 24, 25, 28, 29, 31, 32, 35, 37, 38, and 40 indicate high levels of anxiety as well. Conversely, higher scores on the remaining items of both scales indicate lower levels of anxiety. Each subscale yields a total score ranging from 20 (indicating low anxiety) to 80 (indicating high anxiety). The STAI Form Y has demonstrated strong reliability and validity in the Greek population [13].

Brief Pain Inventory (BPI)

The Brief Pain Inventory (BPI) is a short, easy-to-understand, self-reported questionnaire designed to assess pain. It evaluates both the intensity of pain and the extent to which pain interferes with the patient's daily life and functioning. The BPI also asks about pain relief, pain quality, and the patient's perception of the cause of pain. Patients are asked to rate their current pain and their worst, mildest, and average pain over the past week or the 24 hours preceding the interview. The pain severity is measured using a 0-10 scale, where 0 represents "no pain" and 10 represents "the worst pain that you can imagine." In addition, the questionnaire assesses the degree to which pain interferes with seven areas of the patient's life, including work, activities, mood, enjoyment, sleep, walking, and relationships. These interference items are also rated on a 0-10 scale, with 0 indicating "no interference" and 10 indicating "complete interference." The Brief Pain Inventory (BPI) demonstrates high reliability and validity in Greek people [14].

Data analysis

Quantitative variables were expressed as mean values (SD), while qualitative variables were expressed as absolute and relative frequencies. Pearson’s or Spearman’s correlation coefficients were used to explore the association of two continuous variables. Multiple linear regression analysis in a stepwise method was used with the dependent dimensions of quality of life. The regression equation included terms for demographic characteristics, anxiety (trait and state), and pain (severity and interference). Adjusted regression coefficients (β) with standard errors (SE) were computed based on the results of the linear regression analyses. All reported p-values are two-tailed. Statistical significance was set at p<0.05, and analyses were conducted using IBM Corp. Released 2020. IBM SPSS Statistics for Windows, Version 26. Armonk, NY: IBM Corp.

Ethical considerations

Participants who met the inclusion criteria were informed about the purpose of the study, and confidentiality was assured. Prior to the collection of data, approval was granted from the Institutional Review Board of the Second Health Region of Piraeus and Aegean (Number of approval: 14.06.2024/DAAD 35195). The study was carried out from June 2024 to January 2025. The study was carried out in accordance with the Declaration of Helsinki (1989).

## Results

Sample description

The sample consisted of 100 participants with a mean age of 75.3 years (SD = 7.7 years). Sixty-four percent were women, and almost all (99%) had Greek nationality. Most of the participants (64%) lived in the greater Attica region, 38% were high school graduates, and 41% had either not attended school or had completed primary education. Additionally, half of the participants (51%) were married, and 94% had children.

Moreover, 33% of the participants were living alone, 48.5% lived with their spouse, 28.3% with their children, and none were living in a nursing home. Regarding their employment status, 90% weren’t working and 70% were retired. A total of 37.5% had worked as private-sector employees, 26% were engaged in domestic duties, while 18.8% had worked as public servants, 11.5% were self-employed, and 6.3% were farmers. For 60%, their work had been manual work.

Additionally, 48% described their financial situation as moderate, and 26% as poor. The majority (88%), were insured by a public provider. Almost all (97%) had at least one chronic disease, and more than half (59%) had faced a traumatic event during the last year (Table 1).

**Table 1:** Sample’s characteristics (N=100).

Participant’s characteristics		Ν	%
Gender	Male	36	36.0
	Female	64	64.0
Age, Mean ± SD		75.3 ±7.7	
Residence place	Greater Attica region	64	64.0
	Prefecture capital	22	22.0
	Small town	8	8.0
	Countryside	6	6.0
Educational level	Up to Primary school	41	41.0
	High school - Lyceum	38	38.0
	University or higher	21	21.0
Married	No	49	49.0
	Yes	51	51.0
Children	No	6	6.0
	Yes	94	94.0
Living alone	No	67	67.0
	Yes	33	33.0
Employee	No	90	90.0
	Yes	10	10.0
Was your work manual?	No	38	40.0
	Yes	57	60.0
Describe your current financial situation	Poor	26	26.0
	Average	48	48.0
	Good	22	22.0
	Very good	4	4.0
	Excellent	0	0.0
Insurance	Public	88	88.0
	Private	0	0.0
	Both	4	4.0
	Uninsured	8	8.0
Chronic disease	No	3	3.0
	Yes	97	97.0
Traumatic event	No	41	41.0
	Yes	59	59.0

Chronic diseases of the participants included musculoskeletal diseases (76.3%), hypertension (44.3%), cardiovascular diseases (37.1%), respiratory diseases (21.6%), diabetes mellitus (17.5%), autoimmune disease (14.4%), eye diseases (11.3%), cancer (10.3%), and stroke (5.2%). Additionally, participants’ traumatic events during last year included serious illness/accident (40.7%), death of loved ones (37.3%), debts (27.1%), serious illness/accident of a loved one (23.7%), loss of residence (1.7%), and divorce (1.7%).

Regarding the descriptive statistics of SF-12, STAI, and BPI dimensions, the mean score on the physical dimension was equal to 33.7 (SD=9.8), and on the mental one, 41.7 (SD=11.8). Mean state anxiety was equal to 38.8 (SD =13.1) and trait 43.6 (12.9). Concerning pain dimensions, mean scores of severity and interference dimensions were 4.9 (SD=1.9) and 5.5 (SD=2.3), respectively (Table 2).

**Table 2:** Descriptive statistics of SF-12, STAI and BPI dimensions. SF-12: Short Form-12 Health Survey, STAI: State-Trait Anxiety Inventory, BPI: Brief Pain Inventory

Components of SF-12, STAI and BPI scales	Minimum	Maximum	Mean ± SD
Physical Composite score	18.8	58.6	33.7 ± 9.8
Mental Composite score	17.2	64.9	41.7 ± 11.8
State anxiety	20.0	74.0	38.8 ± 13.1
Trait anxiety	20.0	77.0	43.6 ± 12.9
Severity	0.5	8.5	4.9 ± 1.9
Interference	0.9	10.0	5.5 ± 2.3

Correlation coefficients among quality of life, anxiety, and pain

Both state and trait anxiety were significantly and negatively associated with mental health (rho=-.50, p<.001; r=-.66, p<.001, for state and trait, respectively), indicating that higher anxiety levels (whether trait or state) were associated with poorer mental health quality of life. Additionally, significant and negative were the correlations between pain severity and pain interference with physical (r=-.49, p<.001; rho=-.55, p<.001, for severity and interference, respectively) and mental (r=-.21, p=.035; rho=-.43, p<.001, for severity and interference, respectively) dimensions of quality of life. Thus, greater pain and its interference with daily life and activities were associated with lower quality of life. More severe pain was also associated with higher trait anxiety (r=.26, p=.009), while increased pain interference was associated with both higher state (rho=.25, p=.012) and trait anxiety (rho=.46, p<.001) (Table 3).

**Table 3:** Correlation coefficients between the SF-12, STAI, and BPI dimensions. SF-12: Short Form-12 Health Survey, STAI: State-Trait Anxiety Inventory, BPI: Brief Pain Inventory *p<.05; **p<.01; ***p<.001

Components of SF-12, STAI and BPI scales		Physical Composite score	Mental Composite score	State anxiety	Trait anxiety
Physical Composite score	r/rho		0.22*	-0.18	-0.19
	P		0.028	0.072	0.061
Mental Composite score	r/rho			-0.50***	-0.66***
	P			<0.001	<0.001
Severity	r/rho	-0.49***	-0.21*	0.070	0.26**
	P	<0.001	0.035	0.475	0.009
Interference	r/rho	-0.55***	-0.43***	0.25*	0.46***
	P	<0.001	<0.001	0.012	<0.001

Multiple linear regression analyses with dependent variables the dimensions of quality of life and independent sample’s characteristics, anxiety (state and trait), severity, and interference of pain.

After multiple linear regression analyses were conducted (in a stepwise method), with dependent variables the dimensions of quality of life and independent sample’s characteristics, anxiety (state and trait), severity, and interference of pain, it was found that the interference of pain in daily life and activities, as well as engagement in manual work, were associated with physical health, while trait anxiety, manual work, living alone, and age were associated with mental health.

More specifically, the greater the impact of pain on an individual’s life and functionality, the poorer their physical health (β=-2.438, p<.001). Individuals who had been involved in manual work reported worse physical health compared to those without such work experience (β=-3.625, p=.036). Increased trait anxiety was significantly associated with worse mental health (β=-0.567, p<.001), as well as increased age (β=-0.212, p=.043). Poorer mental health also had those engaged in manual work, compared to those who had not engaged in similar work (β=-6.870, p<.001), and those who lived alone in comparison to those who lived with others (β=-4.420, p=.009) (Table 4).

**Table 4:** Results of multiple linear regression analyses with dependent variables the dimensions of quality of life and independent sample’s characteristics, anxiety (state and trait), severity, and interference of pain. +regression coefficient ± standardized regression coefficient ++standard error

Independent variables	Dependent variables	β+	SE++	b±	P
Physical Composite Score					
	Interference	-2.438	0.371	-0.553	<0.001
	Was your work manual? (Yes vs No)	-3.625	1.703	-0.179	0.036
Mental Composite score					
	Trait anxiety	-0.567	0.060	-0.618	<0.001
	Was your work manual? (Yes vs No)	-6.870	1.593	-0.284	<0.001
	Living alone (Yes vs No)	-4.420	1.666	-0.175	0.009
	Age	-0.212	0.103	-0.137	0.043

## Discussion

The present study showed that increased trait or state anxiety was significantly associated with poor mental health among older adults (rho = -.50, p < .001; r = -.66, p < .001, for state and trait, respectively). A possible explanation for this finding is that anxiety is a complex and multidimensional situation, including subjective discomfort, increased alertness to potential future dangers, avoidance behaviors [15], as well as problems with sleeping and maintenance of concentration, which negatively affect mental health [16]. This finding is congruent with those of Brajer et al. [17], who found that high levels of state and trait anxiety were related to increased levels of depression among older adults [17].

This study also found that increased pain had a significant association with low physical health (β=-2.438, p<.001). This can be attributed to the fact that chronic pain causes low energy, muscle discomfort, limited physical mobility, and difficulty in climbing stairs [18], which leads to reduced functional capacity and increased dependence on performing daily activities [19]. It is important to take into consideration that musculoskeletal pain and reduced physical ability are highly prevalent in the general population [20]. In a similar study, it was found that individuals with persistent pain tended to show reduced physical functioning [21], while in another study pain was correlated with reduced functional performance [22].

Additionally, the results of the present study showed that increased pain interference was associated with higher state (rho=.25 p=.012) and trait anxiety (rho=.46 p<.001). In a similar study, participants who experienced chronic pain exhibited higher levels of anxiety and depression [23]. Similarly, individuals with chronic musculoskeletal pain reported great levels of anxiety, depression, fatigue, and insomnia, while mental health symptoms worsened as the intensity of pain increased [24]. This can also be viewed in the context of long-term work disability, which is associated with fear of pain or reinjury, low expectations of returning to work, and a lack of confidence in handling work-related tasks [20].

Moreover, the findings of this study indicated that individuals who had been involved in manual work reported decreased physical health (β=-3.625 p=.036). In a similar study, lower back problems were common health complaints among construction workers, possibly due to repeated activities involving the lifting of heavy construction materials with improper body posture [25]. It is important to take into consideration that musculoskeletal disorders in the work environment are usually caused by repetitive tasks, uncomfortable postures, strenuous exertions, and extended periods of sitting or standing. These factors can often lead to conditions such as carpal tunnel syndrome, tendonitis, back pain, and neck and shoulder discomfort, resulting in pain, limited mobility, and reduced functionality and affecting individuals’ capacity to effectively fulfill work responsibilities. Thus, low physical health is strongly associated with early retirement among older adults [26].

The results of this study, also, showed that those engaged in manual work had poor mental health (β=-6.870, p<.001). In a similar study, it was found that on-site workers had high workloads, strict deadlines, and increased worries about their physical health and safety. These factors contributed to elevated stress, which negatively affected their job effectiveness [27]. Poor mental health among people with manual work can also be viewed in the context of early retirement since it has been found that decreased work ability leads to high rates of sick leave and early withdrawal from work, which negatively affects mental health [26].

Additionally, the findings of this study indicated that those who lived alone had poor mental health (β=-4.420, p=.009). Similarly, research has shown that increased feelings of loneliness are related to a significant number of depressive symptoms [28,29], while loneliness is strongly associated with a decline in overall mental health [30]. More specifically, social and emotional loneliness is related to a lack of interest, feelings of hopelessness, diminished self-worth, fatigue, and a sense of emptiness [31]. It is important to take into consideration that a major concern of many older adults is their feeling of loneliness, often accompanied by a fear of being abandoned, leading to increased anxiety and fear, which negatively affect their mental health [32].

The present study also showed that high trait anxiety was significantly associated with increased age (β = -0.212, p = .043). This possibly happens because this stage of life is associated with declining health, reduced mobility, loss of partners and close friends, and retirement, which can lead to a diminished sense of identity [33]. Moreover, a common problem occurring in older people is sleep disorders [34], which lead to increased stress, depression, and anxiety [35]. In a similar study with 246 older adults, it was found that older adults who had two or more illnesses experienced increased levels of anxiety and worry [36].

The results of this study offer important information to health professionals about the association between quality of life, anxiety, and pain among older adults. The findings of this study indicate that both state and trait anxiety were strongly associated with poor mental health and quality of life, while increased pain had a strong association with reduced physical health and high state and trait anxiety. Therefore, this study raises the issue of the necessity for early diagnosis of anxiety and pain symptoms in older adults and the provision of the appropriate treatment and psychological interventions in order to improve their overall quality of life. Early diagnosis can be achieved through routine screening in primary care, using validated tools designed specifically for older adults, while training healthcare providers and family members to recognize early symptoms of anxiety and pain is crucial.

Additionally, a multidisciplinary approach to the management of pain can prevent the deterioration of physical and mental health and quality of life among this population. Given the complex biopsychosocial nature of pain, a multidisciplinary approach requires a combination of strategies, including personalized pharmacological treatment, physical therapy to enhance mobility, daily functioning, and resilience, as well as psychological interventions that address emotional, cognitive, and behavioral components of pain.

Limitations of the study

The sample of this cross-sectional study consisted of 100 older adults who were conveniently selected from a single health center in Piraeus. In addition to the non-random sampling method, the sample size was relatively small due to time constraints, which limited the researchers' ability to include other health centers in the Attica region. Furthermore, the cross-sectional design captures data at a single point in time, thereby restricting the ability to draw causal inferences between variables. The use of self-reported questionnaires also introduces potential bias, as these tools rely on subjective assessments. Collectively, these limitations reduce the generalizability of the study’s findings.

## Conclusions

State and trait anxiety were strongly related to poor mental health and quality of life, while increased pain had a strong association with low physical health and high state and trait anxiety. Individuals with manual work had bad physical and mental health, while increased trait anxiety was significantly associated with increased age. Finally, poor mental health was found in people living alone. Thus, health professionals need to have the appropriate training in order to recognize early symptoms of anxiety and pain among older adults. They can also implement personalized pharmacological treatment, physical therapy to improve mobility, as well as psychological interventions in order to improve the quality of their life.
